# Nurse Marion's Romance

**Published:** 1887-04-09

**Authors:** Kate Furley


### Nurse Marion's Romance.

By Kate Furley.

Chapter III.—Jack Holtayne in Hospital.

After feverish nights and dreary days, when his feeble body and fretful mind suffered acutely from every sound, from the splashing of waves and the grinding of machinery, from the gossiping, flirting, and quarrelling of fellow-passengers, till he felt himself an inmate of some floating Hades, it seemed to Jack Holtayne that he had come to a paradise of cool, restful silence when he was laid in his bed at the hospital of St. Lazarus. For the first day or two he received outward impressions but dimly: his contentment was negative, composed of the absence of irritating conditions; and he was too weak and tired to open his eyes and analyse whence this feeling of comfort was derived. He rested from all things, from pain, and exhaustion that was harder to bear than pain; from fretting recollections of past labour and disappointment, and of pleasures that left a gloomier memory than the least successful toil; from everything that could spoil this exquisite Nirvana of peace and calm. The mind was active in his languid frame, but the will had ceased to control it; and it was directed no longer to the hopes and ambitions of the future, but wandered as it would among long-buried half-forgotten scenes, to recall which even in fancy seemed to fill his body with greater strength and his soul with nobler energy than they had known for many a day.

Sometimes he wondered vaguely and lazily if there were indeed some mystic power in native air to sweep away all arid-tainted thoughts of the nearer past, and take him back to his early manhood, when he talked of himself and his future with the sublime vanity and self-confidence of one who has never yet wrestled with the strong power of circumstance, and therefore never doubts the invincibility of his spirit's thew. He could criticise others then, and point out the reason of their failures and mistakes, so easily to be avoided by "himself, the undefeated that should be."

There was a sense of absurdity present to his consciousness now in remembering these aspirations of his youth, and the assured conviction of success that permeated them; yet they had not been so foolish that they could not be shared by at least one, who, half-forgotten long ago in the midst of the world's distractions, seemed almost as soon as he touched English ground to have regained her old gentle influence over him. "It was only a fancy," the consciously active part of his intellect told him, "a dream born of English associations still imperfectly defined and analysed"; but the dream had power over him, and made him murmur, "Marion, Marion," with unconscious lips.

The nurse, bending over him with longing, tender eyes dim with forbidden yet half-happy tears, listened to her name, spoken even in delirium with such weary yearning, and forgave all past pain and injustice for the sake of the love it seemed to express.

"He was angry with me when he acted as he did, but he must have loved me truly, since he has not forgotten me during all these years."

She wove, woman-like, a romance about these ten years of absence, and would not see in the lines of the worn though handsome face upon the pillow, a token of anything meaner than heaven-sent sorrow, bravely borne.

"He married in haste," she said to herself, and made an unwise choice—he was always impulsive, and too ready to believe well of those he met. Evidently his wife has not made him happy. She cannot love him much, or she would not have allowed him to come home alone, so ill as he is."

For it should be observed that Mr. Holtayne had not thought it worth while to send any announcement of his wife's death to his old sweetheart, neither of them being at that moment so much in his mind as a certain venture in opium, which had turned out unsuccessfully.

For the first time since she had taken up the work, Nurse Marion felt that the attention required by the patients in the large ward was troublesome to render. She did not neglect them, but she performed her necessary duties with a shade more of haste than was her wont. She had no time to linger by a bed to utter those words of gentle encouragement, which more than one patient had declared to have effected more than half his cure; and though none of them had any legitimate definite cause of complaint, several felt more or less aggrieved at not seeing so much of their nurse as usual. A strained, anxious look grew upon Marion's face; she became noticeably paler and thinner within a few days; and no wonder, seeing that all the hours she usually took for rest and half of those she gave to sleep were spent by Jack Holtayne's bed.

She knew that though he was very weak and his constitution seemed lacking in recuperative power, his condition was not immediately dangerous. In the case of any other patient, she would have predicted with assurance that Dr. Brandreth's skill and her own care would restore him to health; but in this case anxiety overpowered experience, and every hour she was tortured by the fear that he might die. She watched every sign of conscious life in him with a care as deep and fond as that with which a mother looks for the dawning intelligence of her child, and at every turn of the weary head on the pillow, thought he was arousing from the stupor which enveloped him. She had forgotten the faults that had made her life desolate, and remembered only that he had loved her once.

She was near him when he first opened his eyes with a gleam of comprehension in them. He had not yet fully realised all that had happened, for he expressed no surprise at seeing her, and merely said, "I am glad you are here, Marion. I was dreaming of you."

"And are you pleased to have me near you?" she asked, timidly.

"Yes, of course; you should know that. But, somehow, things seem queer to me. Why, I thought— Is it true, I wonder?"

"What do you mean, Jack?"

"There! to hear you call me by my name makes everything but your presence seem strange; and yet I have an impression that it is that which is strangest of all. I know I'm ill, and that seems to fit in with the impressions I have of the past; but seeing you makes me think them wrong. You might tell me, Marion—and don't think me very stupid for asking—if I have been in India, or if I am only going there?"

"You have been there for ten years, and have just returned."

"I knew it, and yet I didn't believe it when I saw you. I don't understand it at all."

"Don't try to understand it yet," said Marion. "In a day or two, when you are stronger, you will remember everything."

She left him hastily, for she was afraid of betraying an agitation that might injure him; and Holtayne, now that his brain was fully awake, was able very soon to fit into each other the links of the chain of circumstances.

"The hospital is not the best place for you, Mr. Holtayne," said the Doctor next day. "You want purer and more bracing air than London can give. Have you any friends near town to whom you could go?"

"Not one. And I don't want to go away. I have had jolting and worry enough; I need rest now. Besides, I don't want to lose your attendance, nor the nurse's care."

"If you did not go far away—there is a farmhouse near Harrow to which I have sent patients occasionally—I could still attend you."

"And the nurse—Nurse Marion, as you call her—would she come with me?"

"The hospital does send out nurses to private patients; but it is not usual to take them out of the wards if it can be avoided. You could get another."

"I don't want another," said the invalid petulantly. "I like this one; she suits me, and I won't go to the country unless she accompanies me. I would sooner stop here and take the chance of not getting better."

The Doctor hesitated for a moment. To send Nurse Marion away with Holtayne, to let him have sole claim to her care and ministry, to make him the sole occupant of her thoughts, was the last thing he desired to do. But he knew that to anyone in the condition of his patient, little vexations might be serious hindrances to health. He believed, too, more perhaps as a lover than as a physician, that there was not in St. Lazarus' a nurse so skilful and tender as Marion; and forcing his sense of duty to his patient to overcome personal prejudice, he said, "that if it could be arranged," Nurse Marion should be sent to take charge of Holtayne.

What the Doctor said was necessary for a patient, must of course be done, if it was possible. Marion was told of the duty assigned to her, and prepared to fulfil it, without daring to ask herself whether she was grieved or glad that she had it to do.

So the ambulance waggon was brought into requisition, and patient and nurse went out to Harrow, where the Doctor had made preparations for their reception.

"I don't deserve such good fortune as this," said Jack to the nurse when they had been at the farm a few days, and the clear bracing air was bringing back some degree of vigour to his weary frame, though he was still languid and feeble. "After the way in which I left you it is too kind of fate to consign me to your care as soon as I came back to England. That is greater happiness than a lonely man like me has any right to expect."

"But you are not lonely, I hope. Your wife——"

"Ah! did you not know my wife died more than eight years ago?"

"I had not heard of it," answered Marion. She turned abruptly away from him, and began to arrange some flowers in a glass on the window-sill, more willing that the noonday sun should read the secret written on her face than that it should meet her former lover's eye.

His glance followed her, and a slight, well-pleased smile appeared for a moment on his countenance.

"Will you despise me very much, Marion, if I confess that I did not grieve much for her death?" He went on, after a considerable pause: "My marriage was a blunder, entered into with the proverbial haste, and entailing the proverbial result. We were not suited to each other."

"But you——loved each other?" asked Marion, still bending over the flowers.

"I suppose I have no right to say she did not love me, though I think my own thoughts on the subject. I can answer for myself, if it is necessary. But is it? Do you imagine that I had forgotten you within two months of our parting?"

"You seemed to have done so."

"Appearances are deceitful," observed Jack, who seemed to feel the value of proverbial wisdom to-day. "I think you might have guessed the truth," he added, in a tone that conveyed a faint suggestion of offence; "I thought you at least understood my nature. I married because I was utterly lonely and wretched, and perhaps also because I was piqued by your obstinacy. You know it was a mistake, your refusing to marry me when I asked you."

"I could not do otherwise," answered Marion, turning to him with a face on which could still be seen the traces of the long-past struggle between duty and happiness. "My father needed me."

"I needed you as much as he did, perhaps more. You were meant to be the angel of my life, Marion. I could not do without you, and no other could supply your place. But you would not come to me, and the result has been that my life has been such as it would not be good for an angel to see. I would have been a better man if you had married me."

"Don't say that," pleaded Marion, with tears in her eyes. "I cannot even now think I acted wrongly. Even though you did, as you say, require me as much as he, my father had the first claim to my love and care. And yet to think that by staying with him I did harm to you! Oh! it is hard! I thought that I alone suffered by my choice; I can't bear to think that it injured you."

"I'll never hint at such a thing again," Jack promised, having now stated it so plainly that any further hints were quite unnecessary. "After all, I never was worthy of you. I was only a poor ne'er-do-well, who deserved all the hard blows life could give him, and perhaps for your sake I ought to rejoice that you did not share that life with me."

*(To be continued.)*

